# A comparison of self reported air pollution problems and GIS-modeled levels of air pollution in people with and without chronic diseases

**DOI:** 10.1186/1476-069X-7-9

**Published:** 2008-02-28

**Authors:** Fredrik Niclas Piro, Christian Madsen, Øyvind Næss, Per Nafstad, Bjørgulf Claussen

**Affiliations:** 1 Institute of General Practice and Community Medicine, University of Oslo, P.O. Box 1130 Blindern, 0317 Oslo, Norway; 2 Division of Epidemiology, Norwegian Institute of Public Health, P.O. Box 4404 Nydalen, 0403 Oslo, Norway

## Abstract

**Objective:**

To explore various contributors to people's reporting of self reported air pollution problems in area of living, including GIS-modeled air pollution, and to investigate whether those with respiratory or other chronic diseases tend to over-report air pollution problems, compared to healthy people.

**Methods:**

Cross-sectional data from the Oslo Health Study (2000–2001) were linked with GIS-modeled air pollution data from the Norwegian Institute of Air Research. Multivariate regression analyses were performed. 14 294 persons aged 30, 40, 45, 60 or 75 years old with complete information on modeled and self reported air pollution were included.

**Results:**

People who reported air pollution problems were exposed to significantly higher GIS-modeled air pollution levels than those who did not report such problems. People with chronic disease, reported significantly more air pollution problems after adjustment for modeled levels of nitrogen dioxides, socio-demographic variables, smoking, depression, dwelling conditions and an area deprivation index, even if they had a non-respiratory disease. No diseases, however, were significantly associated with levels of nitrogen dioxides.

**Conclusion:**

Self reported air pollution problems in area of living are strongly associated with increased levels of GIS-modeled air pollution. Over and above this, those who report to have a chronic disease tend to report more air pollution problems in area of living, despite no significant difference in air pollution exposure compared to healthy people, and no associations between these diseases and NO_2_. Studies on the association between self reported air pollution problems and health should be aware of the possibility that disease itself may influence the reporting of air pollution.

## Background

Self reported air pollution is sometimes used as a pollution indicator in lack of objective measures. In a recent study by Heinrich et al. [1] on self reported traffic intensity compared to modeled exposure of air pollution from traffic, the subjective assessments of exposure tended to overestimate the modeled estimates of air pollution exposure, indicating only a weak association between self reported and modeled air pollution. The results by Heinrich et al. [1] have important implications for the research on health effects of air pollution, as several studies use subjective assessment of traffic exposure in their efforts to document such effects [2-4]. Additionally, within the literature on area effects on health, composite indexes that include self reported measures of air quality, air pollution or traffic are frequently used [5,6].

A methodological problem arises in studies that have no objectively measured equivalents to their self reported measures of air pollution. Typically, subjects who report on air pollution at their home address also answer health questionnaires. In cross-sectional studies, results could be severely biased if both the exposures and the potential health impacts are assessed subjectively. Studies that rely upon self reported air pollution data face the dilemma of whether their results only express a systematic over-reporting of air pollution among those with the same disease for which they try to establish effects upon from air pollution.

The aim of this article is to explore which factors over and above GIS-modeled air pollution levels that contribute to people's reporting of self reported air pollution problems in area of living. We investigate whether those with respiratory diseases (asthma, chronic obstructive pulmonary disease), other chronic diseases (coronary heart disease, osteoporosis, diabetes and fibromyalgia; a chronic syndrome characterised by diffuse or specific muscle joint, or bone pain, fatigue, and a wide range of other symptoms), or poor self rated health tend to over-report air pollution problems, compared to healthy people.

## Methods

Data were obtained from the Oslo Health Study (HUBRO), a joint collaboration between the Oslo City Council, the University of Oslo, and the Norwegian Institute of Public Health from May 2000 to September 2001. 40 888 persons in five age cohorts were invited (i.e. all inhabitants in Oslo that were either: 30, 40, 45, 60 or 75 years old). Participation rate was 46%. Study population and non-respondents are explained and evaluated in detail elsewhere [7]. In terms of relative effect estimates of disability, the non-participants did not differ from the study participants. Self-selection according to socio-demographic variables had little impact on prevalence estimates. Although unhealthy persons attended to a lesser degree than healthy individuals, social inequalities in health by different socio-demographic variables seemed unbiased. This study also compared the non-attendees in HUBRO with the non-attendees in similar population studies, and concluded that the non-attendance was quite similar to what is usual [7]. The data collection included a main questionnaire, various supplementary questionnaires and a simple clinical examination. Among the 18 770 participants, we have included those who answered the question on self reported air pollution problems, and for whom we had GIS-modeled exposure data of nitrogen dioxide (NO_2_) at their home address (n = 14 294).

### Ethics and approvals

All the participants of the Oslo Health Study gave their written consent. The participants' names and personal ID numbers were omitted when data were used. The Norwegian Data Inspectorate approved the study and the Regional Committee for Medical Research Ethics evaluated it.

### Dependent variable: self reported air pollution problems (APP)

Self reported air pollution problems (APP) was derived from the question "Are you (in your local environment) troubled by air pollution from traffic?", with the response categories 'very troubled' and 'somewhat trouble' collapsed into yes, and 'not troubled' indicating no. There may be some ambiguity in the participants' interpretation of what this question actually measures. Ideally, the participants' answers to this question should be based on a reflection of the local levels of traffic related air pollution levels, which would be a direct way to measure the correspondence between modeled and perceived air pollution. However, the question is formulated so that the participants were asked to rate to what extent they feel troubled by a given level of air pollution. This may vary from person to person, and thus is not the same as considering the amount of air pollution levels *per se*. Instead we consider it an approach that indirectly captures the perceived pollution levels, as the likelihood to feel troubled by air pollution is likely to increase with increased levels of air pollution. The question was chosen from a list of several local items that the participants were asked to consider whether they were troubled by in their local environment (e.g. traffic noise, noise from neighbours, poor drinking water). In this context we believe it was clear to the study participants that the question was related to an assessment of the quality of local area features, not whether or not these features were affecting their health.

### Modeled exposure assessment

Indicator of ambient air pollution exposure at the participants' home addresses was nitrogen dioxide (NO_2_). NO_2 _was chosen as indicator of air pollution exposure as it is considered to be a good marker for traffic related air pollution, has been shown to have spatial variability, and comparisons between modeled and measured levels have shown reasonable agreement [8,9].

Air pollution data was estimated using the GIS-based Air Quality Information System (AirQUIS) developed at the Norwegian Institute of Air Research (NILU) [10,11]. This model combines data on meteorology, emissions, background air pollution concentrations and topography. The model calculates the ambient exposure levels according to home addresses on a km^2 ^grid and a large number of receptor points close to busy roads.

On the day the study participants were examined they were given a supplementary questionnaire in which they should answer the APP-question. The response rate of this questionnaire was 84% (that is 37% of all invited). Most participants returned the questionnaire after 2–4 weeks. Based on this, we calculated the average NO_2 _exposure at the participants' home addresses in the four weeks after examination. This modeled exposure would reflect the time period in which the participants answered the APP-question. NO_2 _values were quintilized for the analyses. Initial analyses with yearly average values of NO_2 _and analyses where NO_2 _was measured continuously were performed with results quite similar to the 4-weeks variable (figures not shown). We therefore chose to proceed with the 4-weeks variable for two reasons. Firstly, to minimize the possibility of migration bias and secondly to better handle the possibility that the participants' reporting of APP could be affected by the air pollution levels at the time of answering the questionnaire, rather than being a reflection of what it is like in the area of living during the year as a whole.

We adjusted for season of examination by categorizing the months of examination into seasons; December, January and February (winter); March, April and May (spring); June, July and August (summer); September, October and November (autumn) [12].

### Independent variables

*Age *was either: 30, 40, 45, 60 or 75 years old. Those aged 40 and 45 were collapsed into one age group. Age and *sex *had no missing values. Missing values for the other variables are described in table 1. *Employment status *was either: fulltime, part-time or not working. *Education *was high (academic college or university education) and low (lower educational forms). *Smoking *was either current smoker, former smoker or never smoker. *Type of dwelling *was either: house (including farms), apartment blocks (including flats in a terraced block of flats and semi-detached houses) or other dwelling types.

**Table 1:** Distributions of independent variables, percentages reporting air pollution problems (APP) and mean NO_2_-levels.

		**N = 14294**			**Mean NO_2_-levels (95% CI) during four weeks after examination**	
**Variables (% Missing)**		**N**	**%**	**%APP**	**APP: No**	**APP: Yes**

Age	*30 years*	3063	21.4	25.9	29.9 (29.4 – 30.4)	36.3 (35.6 – 37.0)
	*40 or 45 years*	5002	35.0	21.6	25.6 (25.2 – 26.0)	33.8 (33.1 – 34.5)
	*60 years*	3569	25.0	20.3	25.4 (25.0 – 25.9)	33.0 (32.1 – 33.8)
	*75 years*	2660	18.6	16.9	26.6 (26.1 – 27.1)	32.4 (31.3 – 33.6)
Sex	*Men*	6355	44.5	20.5	26.9 (26.5 – 27.2)	34.3 (33.6 – 34.9)
	*Women*	7939	55.5	22.0	26.4 (26.1 – 26.7)	33.9 (33.4 – 34.5)
Employment status (0.7%)	*Fulltime*	8426	58.9	21.4	27.0 (26.7 – 27.3)	34.7 (34.2 – 35.3)
	*Part-time*	1506	10.5	22.1	25.1 (24.3 – 25.8)	33.6 (32.3 – 34.9)
	*Not working*	4266	29.8	20.7	26.3 (25.9 – 26.8)	32.9 (32.1 – 33.7)
Education (1.0%)	*Low*	6987	48.9	22.0	27.4 (27.1 – 27.7)	35.0 (34.5 – 35.5)
	*High*	7160	50.1	20.6	25.9 (25.5 – 26.2)	33.1 (32.5 – 33.7)
Smoking (0.7%)	*Never*	6198	43.4	20.6	26.4 (26.1 – 26.8)	34.1 (33.5 – 34.8)
	*Yes, former*	4407	30.8	20.7	26.2 (25.8 – 26.6)	33.5 (32.8 – 34.3)
	*Yes, current*	3582	25.1	22.9	27.5 (27.0 – 28.0)	34.6 (33.8 – 35.3)
Area deprivation (0.9%)	*1st quintile (low)*	3052	21.4	11.2	23.5 (23.1 – 23.9)	30.2 (28.9 – 31.4)
	*2nd quintile*	2891	20.2	26.7	32.2 (31.7 – 32.6)	35.8 (35.1 – 36.4)
	*3rd quintile*	2468	17.3	18.2	23.2 (22.6 – 23.7)	29.1 (27.9 – 30.3)
	*4th quintile*	3249	22.7	17.9	23.5 (23.0 – 24.0)	31.8 (30.7 – 32.9)
	*5th quintile (high)*	2499	17.5	35.3	34.5 (34.0 – 35.0)	38.0 (37.4 – 38.6)
Season of examination	*Winter*	4016	28.1	21.1	30.6 (30.1 – 31.0)	38.3 (37.5 – 39.1)
	*Spring*	3005	21.0	24.1	26.0 (25.5 – 26.6)	33.6 (32.7 – 34.5)
	*Summer*	2286	16.0	21.7	21.4 (20.9 – 21.9)	28.6 (27.7 – 29.5)
	*Autumn*	4987	34.9	19.7	26.2 (25.8 – 26.5)	33.5 (32.8 – 34.1)
Pollution due to wood or oil heating/factory etc. (0.7%)	*No*	13477	94.3	18.1	26.6 (26.4 – 26.8)	33.9 (33.4 – 34.4)
	*Yes*	712	5.0	72.9	27.6 (25.8 – 29.3)	34.9 (33.9 – 35.8)
Type of dwelling (0.3%)	*House/villa*	2960	20.7	13.4	23.9 (23.4 – 24.3)	30.5 (29.3 – 31.6)
	*Blocks*	10541	73.7	22.7	27.1 (26.8 – 27.3)	34.3 (33.9 – 34.8)
	*Other types*	755	5.3	32.8	32.6 (31.5 – 33.7)	37.2 (36.0 – 38.3)
Dwelling damp, draughts or cold (0.6%)	*No*	12750	89.2	19.3	26.5 (26.3 – 26.8)	34.0 (33.6 – 34.5)
	*Yes*	1464	10.2	35.3	27.7 (26.9 – 28.4)	34.2 (33.2 – 35.2)
Poor indoor climate (1.2%)	*No*	13258	92.8	18.9	26.6 (26.3 – 26.8)	34.1 (33.6 – 34.5)
	*Yes*	871	6.1	49.4	27.8 (26.6 – 29.0)	34.5 (33.3 – 35.7)
Depression (2.6%)	*No*	9122	63.8	18.4	26.4 (26.2 – 26.7)	34.2 (33.6 – 34.7)
	*Some*	3710	26.0	26.4	26.7 (26.2 – 27.2)	34.0 (33.3 – 34.8)
	*Yes*	1087	7.6	28.8	27.7 (26.8 – 25.5)	34.0 (32.7 – 35.3)
Asthma (1.8%)	*No*	12732	89.1	20.4	26.7 (26.4 – 26.9)	34.1 (33.6 – 34.5)
	*Yes*	1298	9.1	28.4	25.6 (24.7 – 26.4)	33.7 (32.5 – 34.9)
COPD (2.5%)	*No*	13260	92.8	20.6	26.5 (26.3 – 26.8)	34.1 (33.7 – 34.6)
	*Yes*	678	4.7	34.7	27.6 (26.5 – 28.8)	33.4 (31.9 – 34.9)
CHD (0.8%)	*No*	12969	90.7	20.8	26.7 (26.4 – 26.9)	34.2 (33.8 – 34.6)
	*Yes*	1208	8.5	26.7	26.0 (25.1 – 26.8)	32.5 (31.1 – 33.9)
Diabetes (2.1%)	*No*	13620	95.3	21.2	26.6 (26.4 – 26.9)	34.2 (33.8 – 34.6)
	*Yes*	376	2.6	20.2	26.7 (25.3 – 28.1)	29.6 (26.8 – 32.5)
Fibromyalgia (3.3%)	*No*	12993	90.9	20.6	26.7 (26.5 – 27.0)	34.2 (33.7 – 34.6)
	*Yes*	824	5.8	28.0	24.6 (23.7 – 25.6)	32.6 (30.9 – 34.3)
Osteoporosis (2.8%)	*No*	13331	93.3	21.0	26.7 (26.4 – 26.9)	34.1 (33.7 – 34.5)
	*Yes*	560	3.9	24.3	25.8 (24.6 – 26.9)	32.3 (30.2 – 34.3)
Poor self rated health (1.1%)	*No*	10911	76.3	20.1	26.8 (26.5 – 27.0)	34.5 (34.0 – 35.0)
	*Yes*	3222	22.5	25.7	26.2 (25.7 – 26.7)	32.8 (32.0 – 33.6)

We adjusted for two indicators of housing quality that may affect the likelihood to feel troubled by outdoor air pollution. *Dwelling damp, draughts or cold *was derived from the question "Are you (in your home) troubled by damp, draughts or cold?" with the response categories 'very troubled' and 'somewhat trouble' collapsed into yes, and 'not troubled' indicating no. Similarly, *poor indoor climate *was derived from the question "Are you (in your home) troubled by other forms of poor indoor climate?" with the same responses collapsed. *Self reported pollution *due to wood or oil heating, factory etc. was derived from the question "Are you (in your local environment) troubled by air pollution due to wood or oil heating, factory etc.?" with the response categories 'very troubled' and 'somewhat trouble' collapsed into yes, and 'not troubled' indicating no. This question is rather similar to APP in the sense that there may be some ambiguities in the rationales of the respondents' answers cf. our comments in reference to the APP-question.

*Area deprivation *was a composite measure of five items, a method that has been shown to have stronger independent effects on health than any one variable on its own [13]. The items were area percentages of population affected by social aid, being unemployed, receiving disability pension, having no academic college or university education, and average taxable income in the areas. A rank score for Oslo's 25 administrative areas was calculated and quintilized for the analyses [14]. Each participant was assigned a value for area of living in year 2000. The Oslo City Council provided these data.

In addition, we adjusted for *depression *because there is a possibility that it may be present in some of the chronic diseases and therefore related to the reporting of air pollution. *Depression *was derived from the question "Have you during the last two weeks felt depressed?" with the response category 'no' indicating no, 'a little' indicating some, and the categories 'quite much' and 'very much' indicating yes.

*Asthma, diabetes, fibromyalgia *and *osteoporosis *were derived from the question "Do you have any of these illnesses, or have you suffered from any of them in the past?" *Self rated health *was derived from the question "How would you describe your present state of health?" dichotomised into good (good or very good) and poor (not very good or poor). *Coronary heart disease (CHD) *was measured by The Rose Questionnaire of angina pectoris [15], and *chronic obstructive pulmonary disease (COPD) *by a modified version of the Medical Research Council's questionnaire with three items [16].

### Analyses

The statistical analyses were conducted using the SPSS 11.0 software program. The chi-square test and independent samples t-test were used in comparing the excluded (i.e. those with missing values) and the included respondents. Both logistic and ordinary least square regression was used in the main analyses. In order to investigate a potential over-reporting of APP among people with a chronic disease, we first conducted a logistic regression analysis with APP as the outcome. In this analysis, NO_2 _was included as an independent variable. In a second analysis (ordinary least square regression), NO_2 _measured as a continuous variable was modeled as the outcome (APP was included as an independent variable). The purpose of this was to explore whether health was associated differently with APP and NO_2_. Theoretically, if APP is a good indicator of air pollution levels, then we would expect rather similar associations between health variables and APP/NO_2_-levels. Modelling NO_2 _as the outcome is an unusual strategy, but in this cross-sectional study we were not looking for causal effects, only statistical associations. If health is associated with APP, and not NO_2_, we then believe it indicates an over-reporting of air pollution among people with a disease. An association between health and NO_2_, on the other hand, may provide support for the presence of causal air pollution effects on health. Therefore, in order to test for over-reporting it is important to compare how both APP and NO_2 _are related to health.

In both analyses each health variable was adjusted for age, sex, employment status, education, smoking, area deprivation and season of examination. In the logistic regression analysis we also investigated a model in which dwelling conditions, depression and self reported pollution due to wood or oil heating, factory etc. were removed. The rationale for this was that while we believe these variables are important contributors to the construct of APP, they are poorly measured, and may obscure our results.

## Results

Among the study participants with missing values that were removed from the regression analyses, 23.4% reported APP, whereas 21.3% of those that were included reported APP. The groups did not differ in mean NO_2 _during the four weeks after study conduct (not shown in tables). Mean NO_2 _(95% CI) was 28.6 (28.2 – 29.0) in the missing group and 28.2 (28.0 – 28.5) in the included group. Neither did the groups differ by season of examination. Thus, the missing respondents did not differ by any of the key variables in this study. Remaining results reported here are based on the 12 350 persons that were included in the analyses.

APP increased significantly by levels of NO_2. _7.9% in the quintile with lowest levels of NO_2 _reported APP, 12.3% in the second quintile, 22.6% in the third quintile, 27.5% in the fourth quintile and 33.1% in the fifth quintile (not shown in tables). We divided the participants in two groups, those who reported APP and those who did not (Table 1). APP did not vary much by independent variables, except age (lower among the oldest and higher among the youngest), area deprivation (an uneven distribution but markedly highest among those in the most deprived areas), self reported air pollution due to wood or oil heating, factory etc., and in all three variables representing dwelling conditions. These results corresponded quite well with mean NO_2_-levels, except self reported pollution due to wood or oil heating, factory etc. where there in fact were no significant differences in mean NO_2 _between those who reported that they were troubled by such pollution and those who were not.

There were small variations in APP by season and a larger share of APP in spring than in winter, even though modeled NO_2_-levels were significantly higher in winter. For depression and all health variables (except diabetes) there was a much higher reporting of APP among those with a disease than those without, even though there were no statistically significant differences in NO_2_-levels between the groups. For all variables except diabetes, those who reported APP were exposed to significantly higher levels of NO_2_.

We stratified the participants by season of examination (figures not shown). In all seasons there was an increase in APP with increased levels of NO_2_, even though mean levels of NO_2 _were significantly higher in winter for all quintiles and statistically lower in summer for all quintiles. This indicated that stratification by season was not important as APP did not vary and was verified by stratified regression models showing no differences in associations between independent variables and APP by season (figures not shown).

Table 2 shows results from multivariate logistic regression analysis with associations between asthma and APP, adjusted for all other variables in the model. In the initial model, NO_2 _was strongly associated with APP, with more than five times as big probability for APP among those living in the quintiles with the highest NO_2 _levels compared to those with the least. All quintiles except the two with highest exposures were significantly different from one another. There were also independent associations between other variables (age, employment status and season of examination) and APP. In area deprivation we found that living in the most deprived areas gave an approximately 2.5 times higher probability for APP compared to living in the least deprived areas. Estimates based on season showed that spring and summer were significantly stronger associated with APP than winter. Those who reported asthma had a 51% higher probability for reporting APP.

**Table 2:** Multivariate regression analyses. Odds ratios (95% CI) for APP and Beta-coefficients for NO_2_.

		**Logistic regression analysis** **Dependent variable: APP**		**OLS regression analysis** **Dependent variable: NO_2_**
**Variables**		**Initial model: OR (95% CI)**	**Full model: OR (95% CI)**	**Standardized Beta Coefficients**

Age	*30 years*	1.00	1.00	Ref.
	*40 or 45 years*	1.00 (0.88 – 1.13)	1.01 (0.89 – 1.15)	-.062***
	*60 years*	0.91 (0.79 – 1.05)	1.03 (0.88 – 1.19)	-.054***
	*75 years*	0.54 (0.43 – 0.66)***	0.71 (0.57 – 0.89)**	-.001***
Sex	*Men*	1.00	1.00	Ref.
	*Women*	1.09 (0.99 – 1.19)	1.10 (0.99 – 1.21)	.003
Employment status	*Fulltime*	1.00	1.00	Ref.
	*Part-time*	1.08 (0.92 – 1.26)	1.01 (0.85 – 1.19)	-.022**
	*Not working*	1.40 (1.21 – 1.62)***	1.14 (0.97 – 1.33)	-.038**
Education	*Low*	1.00	1.00	Ref.
	*High*	0.98 (0.88 – 1.08)	0.94 (0.84 – 1.05)	-.018*
Smoking	*Never*	1.00	1.00	Ref.
	*Yes, former*	1.09 (0.98 – 1.22)	1.08 (0.96 – 1.21)	.009
	*Yes, current*	1.00 (0.89 – 1.12)	1.02 (0.90 – 1.16)	.036***
Area deprivation	*1st quintile (low)*	1.00	1.00	Ref.
	*2nd quintile*	2.01 (1.72 – 2.35)***	1.89 (1.60 – 2.23)***	.233***
	*3rd quintile*	1.80 (1.52 – 2.14)***	1.69 (1.41 – 2.02)***	-.020*
	*4th quintile*	1.63 (1.38 – 1.92)***	1.49 (1.25 – 1.77)***	-.002
	*5th quintile (high)*	2.56 (2.18 – 3.02)***	2.13 (1.79 – 2.53)***	.280***
Season of examination	*Winter*	1.00	1.00	Ref.
	*Spring*	1.40 (1.23 – 1.59)***	1.43 (1.24 – 1.64)***	-.147***
	*Summer*	1.45 (1.25 – 1.69)***	1.50 (1.28 – 1.76)***	-.266***
	*Autumn*	1.08 (0.96 – 1.22)	1.10 (0.97 – 1.24)	-.150***
NO_2_	*1st quintile (low)*	1.00	1.00	
	*2nd quintile*	1.69 (1.39 – 2.05)***	1.67 (1.37 – 2.04)***	
	*3rd quintile*	3.07 (2.56 – 3.68)***	2.98 (2.47 – 3.60)***	
	*4th quintile*	3.97 (3.31 – 4.77)***	3.79 (3.13 – 4.59)***	
	*5th quintile (high)*	5.17 (4.31 – 6.20)***	4.90 (4.04 – 5.93)***	
Pollution due to wood or oil heating/factory etc.	*No*		1.00	Ref.
	*Yes*		9.96 (8.18 – 12.30)***	.023**
Type of dwelling	*House/villa*		1.00	Ref.
	*Blocks*		1.41 (1.23 – 1.63)***	.057***
	*Other types*		1.58 (1.26 – 1.99)***	.062***
Dwelling damp, draughts or cold	*No*		1.00	Ref.
	*Yes*		1.35 (1.16 – 1.57)***	-.012
Poor indoor climate	*No*		1.00	Ref.
	*Yes*		2.49 (2.07 – 2.98)***	-.010
Depression	*No*		1.00	Ref.
	*Some*		1.37 (1.23 – 1.53)***	-.009
	*Yes*		1.22 (1.02 – 1.47)*	.001
Asthma	*No*	1.00	1.00	Ref.
	*Yes*	1.51 (1.30 – 1.76)***	1.31 (1.12 – 1.55)**	-.012
APP	*No*			Ref.
	*Yes*			.164***

* p < 0.05, ** p < 0.01, *** p < 0.001

In the full model, where self reported pollution due to wood or oil heating, factory etc., dwelling conditions and depression were included, there were some reductions in the previous significant estimates (employment status was no longer significant), but the associations for quintiles of NO_2 _and APP were still strong, although slightly reduced. The new variables were all significantly associated with APP, indicating that dwelling factors (type of dwelling, damp, draughts or cold in the home and other forms of poor indoor climate) and depression were important for APP. The association between asthma and APP was reduced (odds ratio decreasing from approximately 1.5 to 1.3) but remained significant (p = 0.010). In the OLS regression, most variables (except sex, dwelling conditions and depression) were significantly associated with NO_2_, but not asthma (which was associated with APP).

Table 3 shows odds ratios and standardized beta coefficients for all health variables, when entered one by one into the models in Table 2 (as we did with asthma). Including these health variables did not have any influence on the estimates of the other independent variables in Table 2 (and they are therefore not reported in Table 3). When APP was the outcome in the analysis, all health variables (except diabetes) were significantly associated with APP in the initial model (column a, logistic regression). In the full model (column b, logistic regression), the inclusion of self reported pollution due to wood or oil heating, factory etc., dwelling conditions and depression, led to reduced associations and CHD (p = 0.082) and osteoporosis (p = 0.080) were no longer significant at the 0.05-level. Still, asthma, COPD, fibromyalgia and self reported poor health were all significantly associated with APP. None of the health variables were associated with NO_2 _in the OLS regression analysis in the full model (or in the initial model which we have not reported). Similarly, logistic regression analyses where health variables were treated as outcomes, showed that APP was significantly associated with all outcomes (except diabetes), whereas NO_2 _was not associated with any health outcomes (not shown in tables).

**Table 3:** Individual associations between health variables and APP/NO_2_^1^.

	**Logistic regression analysis** **Dependent variable: APP. OR (95% CI) and p-values.**		**OLS regression analysis** **Dependent variable: NO_2. _Standardized Beta Coefficients (sig.).**
**Variables**	**a) Initial model^2^**	**b) Full model^3^**	**Full model**

Asthma	1.51 (1.30 – 1.76) p = 0.000	1.31 (1.12 – 1.55) p = 0.001	-.012 (.154)
COPD	1.80 (1.48 – 2.21) p = 0.000	1.50 (1.21 – 1.85) p = 0.000	.007 (.392)
CHD	1.43 (1.20 – 1.70) p = 0.000	1.17 (0.98 – 1.41) p = 0.082	-.005 (.513)
Diabetes	1.01 (0.73 – 1.38) p = 0.940	1.03 (0.74 – 1.43) p = 0.831	-.005 (.508)
Fibromyalgia	1.45 (1.19 – 1.77) p = 0.000	1.24 (1.01 – 1.53) p = 0.038	-.015 (.068)
Osteoporosis	1.40 (1.08 – 1.82) p = 0.010	1.27 (0.97 – 1.68) p = 0.080	-.005 (.551)
Poor self rated health (SRH)	1.42 (1.26 – 1.59) p = 0.000	1.19 (1.04 – 1.35) p = 0.008	-.016 (.057)

^1^Adjusted for all variables in table 2. Each health variable entered separately in the model. The associations reported in the model are not adjusted for the other health variables.

^2^The variables included in the initial model were: age, sex, employment status, education, smoking, area deprivation, season of examination, and NO_2_.

^3^The variables included in the full model were: all variables in the initial model and self reported pollution due to wood or oil heating, factory etc., dwelling damp, draughts or cold, poor indoor climate and depression.

## Discussion

We found a strong independent association between GIS-modeled air pollution and self reported air pollution problems, i.e. the higher the levels of air pollution in area of living, the higher the likelihood to report being troubled by air pollution. This is an expected result. The question is rather; what other factors than air pollution itself may contribute in shaping people's perception of air pollution problems in their local neighbourhood? The aim of this study was to investigate whether chronic disease may be one such factor. Independent associations were found between asthma, chronic obstructive pulmonary disease, fibromyalgia and self reported poor health; and APP, whereas independent associations between coronary heart disease and osteoporosis and APP were conditional on the presence of either self reported pollution due to wood or oil heating, factory etc., dwelling conditions or depression.

All associations reported above were independent of age, sex, education, employment status, smoking, season of examination, area deprivation, dwelling type, dwelling conditions and depression. We believe this broad adjustment has adequately excluded the possibility of over-reporting of air pollution problems among people with a disease because of socio-demographic background or a more pessimistic view upon the environments due to residence in a deprived area, and/or poor dwelling conditions and/or depression. We believe our results demonstrate that people with chronic disease tend to over-report air pollution problems, even when the disease is non-respiratory. This is largely due to the fact that none of the health variables were significantly associated with GIS-modeled levels of NO_2_, but they were associated with APP. One interpretation of this, which we doubt, is that such over-reporting of APP may reflect that people with a chronic disease have a lower threshold for being troubled by exposures of daily pollution compared to healthy people. This seems like a convincing argument if only respiratory diseases were considered. But we find it difficult to believe that such an argument is valid in the case of such diseases as fibromyalgia and osteoporosis (partly also CHD and self reported poor health). Another possibility that we doubt, is that the associations between health variables and APP reflect causal air pollution effects on health. This seems unlikely since none of the health variables were associated with NO_2_, and it is again difficult to believe that there should be causal air pollution effects on e.g. fibromyalgia and osteoporosis.

The strong associations that were found between all diseases except diabetes and APP, and the lack of such associations between the same diseases and NO_2 _indicates a general tendency of over-reporting, rather than a lower threshold for being bothered by air pollution among people with a disease. These findings also demonstrate an insufficient correspondence between APP and NO_2 _which makes APP an unreliable indicator of air pollution.

### Study limitations

There are several limitations to our study. Our health measures were self reported. Thus we cannot say whether our results indicate that those with a disease over-report air pollution problems or whether those likely to report disease in a questionnaire are also over-reporting air pollution problems.

We were unable to account for two conditions that may be important. First, *daily mobility*, in order to capture some of the potential for exposure misclassification in those that reside in the city but work elsewhere as those who work close to their homes would be most likely to have an accurate exposure assessment [17]. Second, objective measures of *dwelling conditions*, as our data on these aspects were self reported and may be biased by disease. Previous studies have found that those who rent (compared to those who own) their dwellings are significantly more affected by noise, hazards, vibration, cold and dampness [18]. Indoor exposures may be as important as outdoor exposures [19], although there is evidence that indoor pollution can be directly related to outdoor pollution [20]. Other dwelling factors that righteously may shape reporting of air pollution problems (floor level, whether windows are against road or backyard, etc.) were not available.

Thus, APP is a concept that reflects much more than just exposure to different levels of air pollution, e.g. NO_2_. It may therefore not be a very good agreement between the two. We identified several other factors that were independently associated with APP, but not with NO_2._Interestingly, we found that the exclusion of some of the dwelling factors led to increasingly stronger associations between health variables and APP, which indicates that APP does not only reflect air pollution in the local area, but may also reflect air *quality *in the home itself.

The items we used to measure dwelling factors (damp, draughts, cold or poor indoor climate), as well as our item measuring depression, are of some concern. They are all self reported, not validated, and are somewhat ambiguous. In lack of better data, we still considered it useful to include them in our analyses, as they are intended to measure assumingly important contributors to APP. Our analyses proved this to be right. These variables were independently associated with APP, and they did not attenuate any other associations except the health variables. This indicates that they capture some aspects that are important for the reporting of APP. This became especially clear when we analysed their associations with NO_2 _and found that none of them were significantly associated.

### Contributors to the construct of self reported air pollution problems

Different environmental impacts may result from at least two important pathways: differential exposures or differential susceptibilities [21]. The incorporation of social stratification as a health effect modifier is well established in air pollution epidemiology [17,22-24]. Evidence have been provided that lower socio-economic status areas [25] and households [19] experience the worst air quality, described as examples of the 'inverse air law' [19], analogous to the inverse care law, i.e. that people with the worst lung function tend to live in areas with the worst air quality. This may trigger 'the triple jeopardy', which begins with increased exposure among lower status groups, is augmented by the pre-existing burden of poor health that accompanies low status, and is confounded by an interaction between the two conditions [26].

Over and above modeled air quality many aspects of the environment have been identified in shaping people's perception of the air quality [27-29], indicating that perceptions are socially and culturally constructed [30]. Individuals' perceptions of whether or not they have the ability to bring about change through their behaviour may also influence the perception of local air quality or degree of air pollution problems [27]. For example, when the analyses of Heinrich et al. [1] were restricted to participants with asthma or hay fever, the subjective assessments of air pollution from traffic were increasingly overestimated compared to the modeled levels. Over-reporting of pollution exposure among people with respiratory disease (or any family members) has been found in other studies [31,32], and it has been argued that this over-reporting could be caused by the publicity/media coverage given to air pollution and respiratory health [31]. Therefore, it does not seem unreasonable to claim that the associations between self reported air pollution/traffic and disease may be severely biased by the concern and awareness of having a disease that the lay public link to air pollution. For example, Petrie et al. [33] identified how certain modern health worries were associated with having a disease that most people would be likely to associate with it (e.g. tainted food concerns and gastrointestinal problems, or toxic intervention concerns and pseudoneurology complaints). Seemingly, this logic does not apply to people with diabetes, in which social inequalities are not so marked [34]. The distribution is less stratified by social position and area of living in Oslo, compared to other diseases. The patients themselves often regard their condition as partly a result of obesity, but not from social circumstances, and not from pollution which is more prevalent in the deprived areas of Oslo. Thus, they may be less apt than other chronically ill to be concerned about pollution [34].

It has been claimed that more educated people have longer time horizons than the poorly educated [35], which could explain why well-educated people behave differently as they are more concerned about (and possibly more aware of) the long-term consequences of day-to-day activities [36]. It could be that those with diseases commonly related to air pollution are more likely to report air pollution problems due to a greater concern of the long term-consequences of such exposure. This assumption was tested in our analyses by including both respiratory and non-respiratory diseases, and our conclusion is that the over-reporting of air pollution problems seen for all diseases indicates an over-reporting from chronic disease in general, and not only from those diseases commonly related to air pollution.

The association between air pollution and respiratory health seem well established [37,38], although there is unclear evidence of impacts of outdoor air pollution and asthma incidence [38-40]. Thus, claiming that self reported air pollution/traffic intensity is predictive to health seems plausible. But such a presumption is normally not tested against diseases for which the etiological evidence in reference to air pollution is scarce or missing. This is especially problematic in those studies that do not have any objectively measured equivalents to the self reported problems, as they are then unable to differentiate the part of the association into that of true exposure and that of a socially or culturally constructed bias. Three such biases were tested in our analyses; that of disease, that of living in deprived areas, and that of depression. After the partitioning of NO_2_and disease/area deprivation/depression was done in our analyses, our results indicated, despite the cross-sectional design, that health status affects the reporting of air pollution problems, making the latter an unreliable variable in studies on air pollution effects on health.

## Conclusion

Modeled air pollution and self reported air pollution problems are strongly associated. However, several other factors than air pollution itself contribute to people's reporting of air pollution problems. Independent associations between both respiratory and non-respiratory diseases and self reported air pollution problems were found after adjustment for several socio-demographic variables and GIS-modeled air pollution, whereas no such associations were found between diseases and NO_2_. We therefore believe that people with a disease are in general more likely to report air pollution problems, regardless of the pollution they are in fact exposed to. This is a methodological problem, which should be carefully considered in studies that either tries to find associations between air pollution and health using self reported pollution or traffic intensity, or in studies that try to establish a predictive effect of perceptions of neighbourhood problems on health.

## Competing interests

The author(s) declare that they have no competing interests.

## Authors' contributions

FNP planned the study design, performed the analyses and wrote the manuscript. CM and PN contributed to the acquisition of data and preparing the data files, planning the study design, contributed with academic discussions and drafted and revisited the manuscript. ØN and BC participated in planning the study design, contributed with academic discussions and drafted and revisited the manuscript. All authors read and approved the final manuscript.
